# Long-term, high frequency *in situ* measurements of intertidal mussel bed temperatures using biomimetic sensors

**DOI:** 10.1038/sdata.2016.87

**Published:** 2016-10-11

**Authors:** Brian Helmuth, Francis Choi, Allison Matzelle, Jessica L. Torossian, Scott L. Morello, K.A.S. Mislan, Lauren Yamane, Denise Strickland, P. Lauren Szathmary, Sarah E. Gilman, Alyson Tockstein, Thomas J. Hilbish, Michael T. Burrows, Anne Marie Power, Elizabeth Gosling, Nova Mieszkowska, Christopher D.G. Harley, Michael Nishizaki, Emily Carrington, Bruce Menge, Laura Petes, Melissa M. Foley, Angela Johnson, Megan Poole, Mae M. Noble, Erin L. Richmond, Matt Robart, Jonathan Robinson, Jerod Sapp, Jackie Sones, Bernardo R. Broitman, Mark W. Denny, Katharine J. Mach, Luke P. Miller, Michael O’Donnell, Philip Ross, Gretchen E. Hofmann, Mackenzie Zippay, Carol Blanchette, J.A. Macfarlan, Eugenio Carpizo-Ituarte, Benjamin Ruttenberg, Carlos E. Peña Mejía, Christopher D. McQuaid, Justin Lathlean, Cristián J. Monaco, Katy R. Nicastro, Gerardo Zardi

**Affiliations:** 1 Northeastern University, Marine Science Center, 430 Nahant Rd., Nahant, Massachusetts 01908, USA; 2 The Downeast Institute, Beals, Maine 04611, USA; 3 University of Washington, School of Oceanography, Seattle, Washington 98195, USA; 4 University of California, Davis, Department of Wildlife, Fish, and Conservation Biology, Davis, California 95616, USA; 5 University of South Carolina, Department of Biological Sciences, Columbia, South Carolina 29208, USA; 6 W.M. Keck Science Department of Claremont McKenna, Pitzer and Scripps Colleges, Claremont, California 91711, USA; 7 Scottish Association for Marine Science, Oban, Argyll PA37 1QA, Scotland; 8 Anne Marie Power, School of Natural Sciences, National University of Ireland Galway, Galway H91 TK33, Ireland; 9 School of Life Sciences, Galway-Mayo Institute of Technology, Galway H91 T8NW, Ireland; 10 Marine Biological Association of the United Kingdom, Plymouth, Devon PL1 2PB, UK; 11 University of British Columbia, Department of Zoology and Biodiversity Research Centre, Vancouver, British Columbia, Canada V6T1Z4; 12 University of Washington, Department of Biology, Seattle, Washington 98195, USA; 13 Oregon State University, Department of Integrative Biology, Corvallis, Oregon 97331, USA; 14 University of California, Davis, Bodega Marine Reserve, Bodega Bay, California 94923, USA; 15 Centro de Estudios Avanzados en Zonas Aridas, Coquimbo 1780000, Chile; 16 Stanford University, Hopkins Marine Station, Pacific Grove, California 93950, USA; 17 University of Waikato, Environmental Research Institute, Tauranga 3110, New Zealand; 18 University of California Santa Barbara, Marine Science Institute, Santa Barbara, California 93106, USA; 19 Universidad Autónoma de Baja California, Instituto de Investigaciones Oceanológicas, Ensenada, Baja California 22860, Mexico; 20 Rhodes University, Department of Zoology and Entomology, Grahamstown 6140, South Africa

**Keywords:** Ecophysiology, Marine biology, Climate-change impacts

## Abstract

At a proximal level, the physiological impacts of global climate change on ectothermic organisms are manifest as changes in body temperatures. Especially for plants and animals exposed to direct solar radiation, body temperatures can be substantially different from air temperatures. We deployed biomimetic sensors that approximate the thermal characteristics of intertidal mussels at 71 sites worldwide, from 1998-present. Loggers recorded temperatures at 10–30 min intervals nearly continuously at multiple intertidal elevations. Comparisons against direct measurements of mussel tissue temperature indicated errors of ~2.0–2.5 °C, during daily fluctuations that often exceeded 15°–20 °C. Geographic patterns in thermal stress based on biomimetic logger measurements were generally far more complex than anticipated based only on ‘habitat-level’ measurements of air or sea surface temperature. This unique data set provides an opportunity to link physiological measurements with spatially- and temporally-explicit field observations of body temperature.

## Background & Summary

Increasingly, researchers are emphasizing the need to consider physiological mechanisms when forecasting the effects of global climate change on organisms and ecosystems^1–3^. Specifically, studies have highlighted a need to understand how environmental conditions vary in space and time^4^ in addition to the details of how organisms respond to those variables^5–8^ as a means of evaluating inter- and intraspecific vulnerability (‘winners and losers’)^9,10^, the probability of invasion by non-native species^11,12^, changes in patterns of abundance and distribution^13,14^, and declines in biodiversity^15^ and ecosystem services^16^.

Notably, there is concern that simple correlations between environmental measurements (such as air, land surface and sea surface temperature) and species distributions may fail under the novel conditions presented by climate change^17^, highlighting the need to extrapolate from experiments conducted under controlled conditions to projections of future climate impacts^3,18^. There has also been an emphasis on considering the cumulative impacts of physiological stress^14,19^ on patterns of growth^20^ and reproduction^21^ rather than focusing solely on lethal extremes^19^.

However, making connections between the lab and field can be far more complex than is often assumed^4^. For example, a number of theoretical and empirical studies have explored the often over-riding importance of spatial and temporal variability in environmental parameters^9,22^, which is not captured when experiments are based only on monthly, yearly or decadal averages^23,24^. Moreover, while large-scale measurements of environmental conditions made by satellites, buoys, and weather stations provide critical insights into rates of environmental change on large scales^25^, at a proximal level these habitat-level measurements may not always serve as good indicators of physiological stress^4,26^. In fact, the only ‘environmental signals’ that matter to an organism are those that the organism actually experiences^27^. Making connections across scales that span from organismal to biogeographic is no easy matter, but is crucial if we are to effectively forecast ongoing responses to environmental change^28,29^.

One of the most obvious examples of the complex ways climate defines weather patterns, and weather then drives niche-level organismal responses^30^, is how climate change is ultimately reflected as changes in plant and animal body temperatures. The vast majority of organisms on Earth are ectothermic poikilotherms, so that their body temperatures and thus levels of physiological performance change with ambient environmental conditions. For terrestrial and intertidal ectotherms (and even some shallow-water corals^31^), body temperatures are driven by multiple environmental parameters, most notably solar radiation, air and water temperatures and wind speed^32–34^. The structure of an organism’s microhabitat, and especially its exposure to direct solar radiation, can have enormous implications for its body temperature, such that animal temperatures are only close to air temperature in fully shaded microhabitats^26,35^. While many animals can behaviourally select among these microhabitats as a means of thermoregulation^36^, others are functionally sessile and thus have body temperatures determined by very local topography. To further complicate matters, the size, morphology and colour of organisms, as well as their ability to form aggregations^37,38^ can affect heat exchange so that two organisms exposed to identical microclimatic conditions can have very different body temperatures^39,40^. To contend with these issues, multiple authors have developed heat budget models that factor-in the characteristics of the organism^26,33,41^ to predict body temperatures using weather data as inputs.

An alternative approach—and one that is required to validate biophysical (heat budget) models—is to use *in situ* sensors specifically tailored to record temperatures relevant to the organism being studied, either directly or through the use of biomimics^42^. Biomimetic sensors (biomimics) match the thermal characteristics (size, morphology, colour, material properties) of living organisms^43,44^, serving as an effective tool for recording organismal body temperature in their natural environment^45,46^. Here we report on a long-term data set of temperatures recorded by biomimetic loggers thermally matched to bivalves (mussels) in the intertidal zone, one of the most physiologically harsh habitats on Earth. Over the course of a 24-hr period, intertidal animals and algae are alternately exposed to water at high tide and to air, wind and solar radiation at low tide. Thus, their temperature not only depends on local weather conditions but also on the timing and duration of low tide^47^. We have previously shown, for example, that consistent differences in the timing of low tide relative to high levels of solar radiation create geographic mosaics in low tide temperature, where mussel body temperatures at higher latitude sites can be much higher than those at low latitude sites^40,47,48^. As ecosystem engineers^49^ mussels in particular have a large influence on the stability and biodiversity of the intertidal community and so quantifying their survival and physiological performance has significant ecosystem-level consequences^50,51^.

## Methods

We used biomimetic loggers to estimate temperatures of the mussels *Mytilus californianus* (West coast of North America), *M. edulis* and *Geukensia demissa* (East coast of North America), *M. chilensis* (Chile), *Perna perna* (South Africa) and *P. canaliculus* (New Zealand). We also deployed unmodified commercial loggers directly on rock surfaces at multiple sites (Australia, Ireland, Mexico, Scotland, U.K., U.S.) that recorded temperatures relevant to barnacles, newly settled mussels and other organisms that are sufficiently small that their temperatures mirror those of the underlying rock^52^.

**Figure 1:**
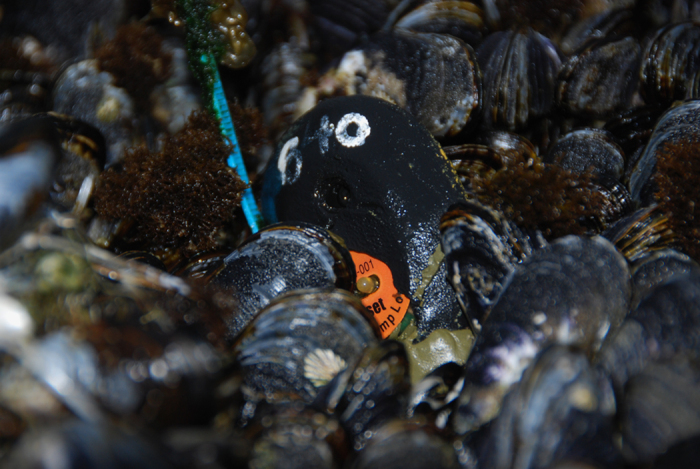
Epoxy ‘robomussel’ biomimetic logger (~8 cm in length) deployed in growth position in a *Mytilus californianus* bed. Loggers were designed to match the thermal characteristics of bivalves and were typically made of epoxy (as shown) but real shells filled with silicone were also used, especially for smaller (4 cm) mussels.

Each biomimetic sensor (‘Robomussel’; Fig. 1) consisted of either a commercially-available TidbiT logger (TB132-20+50 and UTB1-001; Onset Computer Corporation, Pocasset, MA) encased in black-coloured polyester resin (Evercoat Premium Marine Resin, Illinois Tool Works, Inc.), or a real mussel shell filled with silicone and encasing a Tidbit or a Thermochron iButton logger (DS1922L-F5; Maxim Integrated, San Jose, California). Both instruments are factory calibrated: Tidbit loggers have a reported accuracy of 0.21 °C and a stability (drift) of 0.1 °C per year (http://www.onsetcomp.com/products/data-loggers/utbi-001) and ibuttons have an accuracy of 0.5 °C (https://datasheets.maximintegrated.com/en/ds/DS1922L-DS1922T.pdf); the drift is reported by the manufacturer to be negligible, especially when compared to the ~2 °C accuracy of the biomimic loggers (see *Technical Validation* below). Because of loss due to waves, each logger was typically used for only 2–3 years. Details on logger designs and field tests are described in detail in previous publications^44,45,53^. In brief, logger thermal characteristics were calculated using empirical measurements of shell and tissue mass against length from adult *Mytilus californianus* collected on the west coast of North America. In addition to morphology (which determines convective heat flux) and colour (which affects solar heat load), the primary consideration is the maintenance of thermal inertia (the tendency of an object to resist temperature change as a function of external forcing). Mass/length relationships were combined with measurements of the specific heat capacity of shell and tissue to estimate total thermal inertia as a function of size^45^. This was then compared to the thermal mass of polyester resin mussels of different lengths. The point where the two curves intersect is~8 cm shell length; this was the size of the epoxy loggers. Silicone molds were cast from a representative 8 cm mussel, and were in turn used to pour two-part polyester resin (Evercoat) around the commercial TidbiT logger.

In some cases, iButton loggers were encased in ~8 cm mussel shells filled with silicone, which has a mass*specific heat similar to that of water. Comparisons of these instruments against adjacent mussels showed that silicone-filled shells recorded temperatures within ~1 °C of living animals^54^. However, these loggers were considerably less durable and required more frequent maintenance (~bimonthly) than epoxy mussels (every 6–10 months), and so were used only infrequently at most sites. At some sites where the targeted mussel species is smaller (e.g., *M. edulis* in the Gulf of Maine), we used 4 cm mussel shells. Loggers of differing size were never used at the same site, and are distinguished from one another in the database. Nevertheless, any direct comparison between data collected by loggers of different sizes should be made with caution, as size can affect mussel temperature by several degrees^55^.

Robomussels were deployed primarily on hard rock substrate, in growth position (posterior upward) in intact beds using Z-spar splash zone epoxy putty (Fig. 1). Care was taken to ensure that the logger was completely surrounded by other mussels, as tests showed that loggers deployed as solitary individuals tended to yield anomalously high readings. On the east coast of North America, loggers were also deployed at soft sediment (marsh) sites in mud substrate by attaching the loggers to dowel rod.

**Figure 2:**
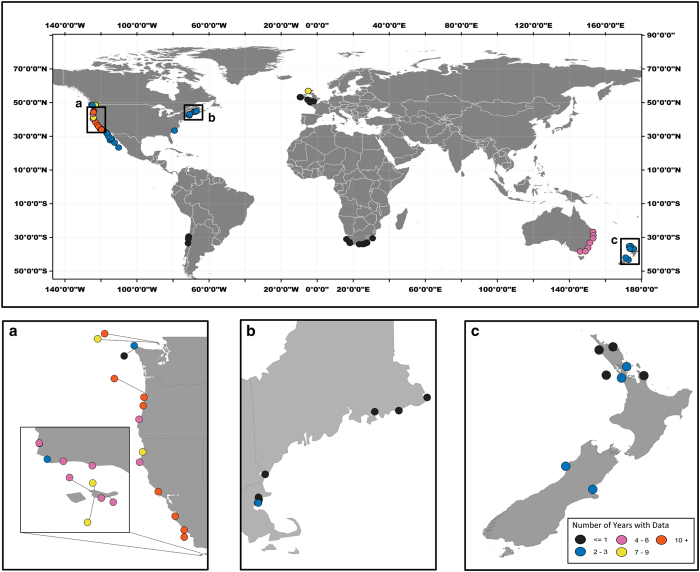
Map of logger deployment sites. Colors indicate approximate length of deployment, which ranged from one or two seasons to almost 18 years. Insets show (**a**) West and (**b**) East coasts of the United States and (**c**) New Zealand.

Deployment began in 1998 at the Hopkins Marine Station in Pacific Grove, California^54^, and was expanded to other sites beginning in 2000 (Table 1 (available online only), Fig. 2). Total deployment time varied by location, ranging from less than a year to almost 18 years (average deployment time of 4 years). The number of loggers deployed and lost due to wave dislodgement also varied at each site, but a standard protocol was to deploy at least 3 loggers in the middle of mussel beds on horizontal, unshaded surfaces. At most sites, loggers were deployed at the upper edge of the mussel bed (‘upper’), half way between the upper and mid levels (‘upper mid’), mid level (‘mid’), half way between the mid and lower edge of the bed (‘lower mid’) and at the bottom of the mussel bed (‘lower’).

Loggers were programmed to record at intervals of 10–30 min and left in the field for periods up to 9 months before they were removed for downloading, and replaced with another logger. Every effort was made to place this new logger in precisely the same position in the bed as the logger being retrieved. All logger clock times were set to GMT. In the U.S., the absolute tidal elevation (height above chart datum) was measured with a Trimble R8 GNSS GPS system capable of sub-cm resolution. Temperature records were also used to record wave swash by comparing sudden drops in temperature (an indication of first wave splash following exposure at low tide) against predicted tidal elevations. The measurements of ‘Effective Shore Level’ can subsequently be compared against buoy records of significant wave height in order to estimate wave splash as a function of nearshore wave height at each site^56,57^.

### Code availability

Code written in R^58^ was used to trim data recorded by each logger before and after deployment. A separate software program (SiteParser) is also available on the Northeastern website to determine the incidence of wave splash^56,57^. This is accomplished by comparing rapid (user-defined) drops in temperature, indicative of the return of the tide, against predicted (Xtide software, www.flaterco.com/xtide) or measured (tidesandcurrents.noaa.gov) tide height for each site. By comparing these measurements against measured logger tidal elevations, it is possible to calculate the ‘effective shore level’ of a logger as a function of nearshore wave height^56^. This also provides a method of dividing logger temperatures into aerial and submerged records. Notably, the choice of temperature drop determines both the accuracy of the division between aerial and submerged records, as well as the total amount of data available. Specifically, the choice of a larger temperature drop tends to increase certainty as to temperature divisions, but can restrict the amount of data to days when such drops are observed. For this reason, the database provides data that have not been analyzed in this manner, but instead provides tools for the user to do so. A link to the open source SiteParser software program is provided on the Northeastern database website, along with links to all metadata including (when available) logger elevations.

## Data Records

**Figure 3:**
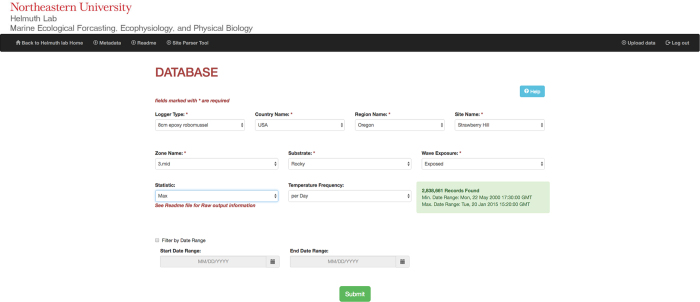
Northeastern database showing dropdown menus. Users select Biomimic type (e.g., 8 cm epoxy logger); Country and Region (e.g., state); Site name; Intertidal zone (e.g., upper, mid, lower); Substrate type; Wave exposure, and Data statistic (raw, mean, maximum, or minimum over ranges of daily, monthly or yearly).

Data from all loggers are archived in two databases. The first is a searchable database maintained by Northeastern University (www.northeastern.edu/helmuthlab/Research/Database.html) and provides unrestricted access to data as well as to associated links such as the SiteParser software described above. Metadata for each microsite are included as a downloadable spreadsheet, which includes, for each site: Country, Region, Site name, and GPS coordinates (Table 1 (available online only)). The metadata file also includes information specific to each microsite, including: Biomimic logger type (unmodified ibutton, unmodified TidBit, epoxy [8 cm] mussel logger, shell (silicone-filled) mussel logger [4 or 8 cm length]), Substrate (rocky, muddy, tidepool), Tidal elevation zone (low, lower mid, mid, upper mid, or upper), Wave exposure (protected or exposed), and Start and end dates (Table 2). At the Northeastern website, data can be viewed and downloaded using a series of drop-down menus (Fig. 3). Given the range of selections, the database provides the range of dates over which data meeting those criteria are available (this information is also included in the metadata file). Data from each logger can be downloaded as raw data, as well as daily, monthly or annual maxima, minima and averages. Note that data include both aerial and submerged temperatures, but raw data can be parsed using the software provided. In instances where multiple microsites meet the selected criteria, the program takes the average at each time point from the maximum number of loggers available before calculating summary statistics. Data from all microsites can be downloaded as raw data to avoid this averaging procedure.

Raw data in text file format as well as associated metadata are also archived in a public repository (Data Citation 1). Files are organized in to a series of subfolders organized by Country, Region and Site (Table 1 (available online only)). Metadata identical to those available at the Northeastern site are also included as a downloadable file. Each data file contains information specific to the microsite in its header, and follows a 10 letter/6 number naming convention as follows: BM (indicating biomimetic logger database); Logger type (RM for mussel loggers [‘Robomussels’] or RB for unmodified loggers [‘Robobarnacles’]); 6 letter site code (Table 1 (available online only); Country, Region, Site); two-digit microsite ID and four digit Year.

## Technical Validation

Comparisons of logger temperatures against tissue temperatures of adjacent live mussels made using thermocouples are presented in four publications^44,45,54,59^. The first compared temperatures recorded by a thermistor with the tip embedded in a silicone-filled shell against point measurements made from adjacent mussels in the field in Pacific Grove, California and found an average difference of ~0.75 °C (ref. 54). The second involved a more comprehensive set of tests of epoxy (polyester) loggers in both the field and in a wind tunnel fitted with a heat lamp^45^. In the laboratory experiments, the average difference between loggers and live mussels in artificial beds was ~2.2 °C (ref. 45). Notably, the average difference between live mussels and unmodified loggers (TidbiTs) in the same experiment was 14.6 °C. Field-tests yielded similar results, with an average error of 2.7 °C between robomussels and live mussels^45^. A follow-up study with additional laboratory tests over a wider range of temperatures (10–50 °C) reported a Root Mean Square Error (RMSE) of 3.84 °C with a correlation coefficient of 0.89 between loggers and live mussels, with a bias of 0.8 °C where loggers tended to overestimate temperatures slightly under extreme conditions^44^. Finally, iButton loggers placed in the middle of silicone-filled *Geukensia demissa* shells were tested in a wind tunnel in artificial beds under a range of wind speeds; results showed average differences of ~1.0–1.5 °C (ref. 59).

## Usage Notes

**Figure 4:**
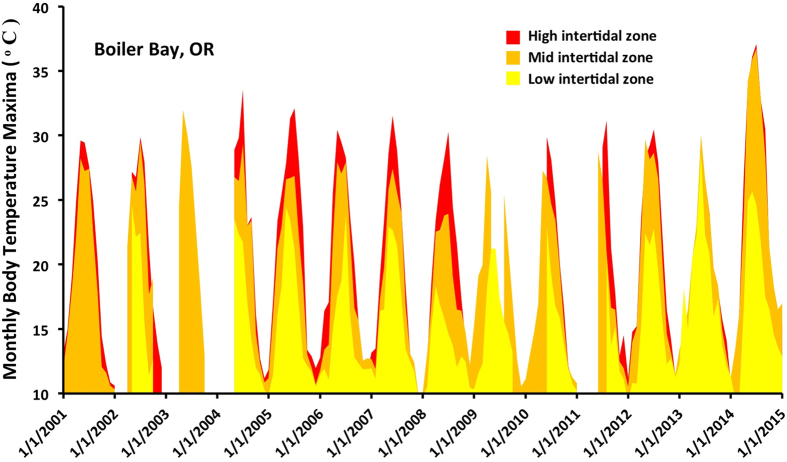
Monthly average daily maximum temperature at low, mid and upper intertidal elevations at a relatively wave-protected bench in Boiler Bay, Oregon.

**Figure 5:**
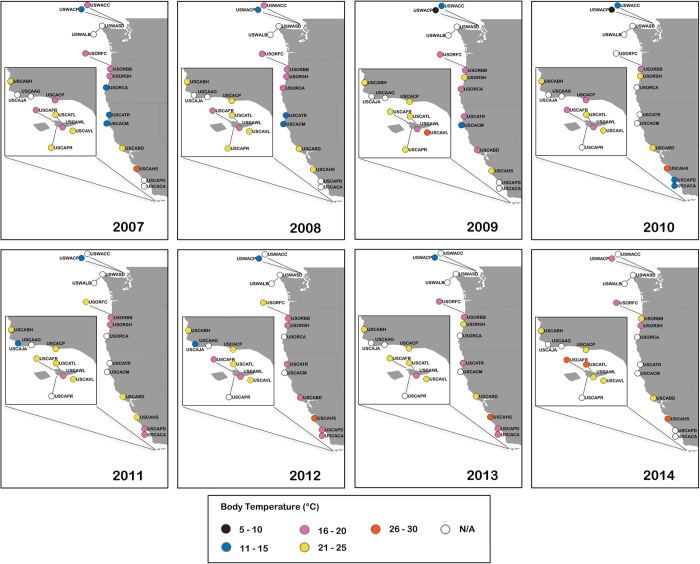
Monthly average daily maximum temperature (for the hottest month of each year at each site) at mid intertidal elevations along the west coast of the United States (2007–2014).

Portions of the logger data presented here have been used in multiple field studies, and have provided context for laboratory studies. At small scales, biomimetic loggers (both loggers that we deployed as well as similar loggers made by other researchers) have been used to record differences in temperature among microhabitats (shaded and unshaded surfaces) and tidal elevations (Fig. 4) and the results compared to measurements of biochemical indicators of stress such as heat shock proteins^54,60^, gene expression^61^, reproductive condition^62^, and to the fine-scale distribution of native and non-native species^63^. At biogeographic scales, robomussels have been used to document thermal mosaics across large latitudinal gradients^40,48^ (Fig. 5) and the results related to patterns of mortality^64^, physiological stress^65–67^ and growth^68,69^, as well as interspecific differences in physiological stress^39^ and geographic distribution^70^. Measurements from mussel biomimetics have been used to test heat budget models that estimate animal temperature using data from weather stations and satellites^71–73^. Robomussels have also been used as part of controlled laboratory experiments that strive to replicate realistic field conditions^37,74^. Finally robomussel data can be used to estimate wave splash and water temperature^56,57^, although in this regard they do not present a major advantage over unmodified loggers.

## Additional Information

**How to cite this article:** Helmuth, B. *et al.* Long-term, high frequency *in situ* measurements of intertidal mussel bed temperatures using biomimetic sensors. *Sci. Data* 3:160087 doi: 10.1038/sdata.2016.87 (2016).

## Supplementary Material



## Figures and Tables

**Table 1:** Metadata describing sites where loggers were deployed and type of biomimetic sensor deployed

**Site Code**	**Site name**	**Region**	**Country**	**Logger type**	**Latitude**	**Longitude**
AUNSBE	Bermagui	New South Wales	Australia	Tidbit	−36.4229	150.0824
AUNSCB	Cape Byron	New South Wales	Australia	Tidbit	−28.6337	153.6382
AUNSGB	Garie Beach	New South Wales	Australia	Tidbit	−34.1734	151.0646
AUNSHP	Haycock Point	New South Wales	Australia	Tidbit	−36.9501	149.9416
AUNSKI	Kiama	New South Wales	Australia	Tidbit	−34.6649	150.8557
AUNSMR	Mimosa Rocks	New South Wales	Australia	Tidbit	−36.5842	150.0510
AUNSPM	Port Macquarie	New South Wales	Australia	Tidbit	−31.4620	152.9366
AUQLNH	Noosa Heads	Queensland	Australia	Tidbit	−26.3793	153.1026
AUVICP	Cape Paterson	Victoria	Australia	Tidbit	−38.6743	145.6331
AUVIKI	Kilcunda	Victoria	Australia	Tidbit	−38.5519	145.4669
AUVIMA	Mallacoota	Victoria	Australia	Tidbit	−37.5734	149.7659
CABCSI	Seppings Island	British Columbia	Canada	8 cm epoxy Robomussel	48.8391	−125.2076
CLCOTE	El Temblador	Coquimbo	Chile	8 cm epoxy Robomussel	−29.5000	−71.3200
CLCOGU	Guanaqueros	Coquimbo	Chile	8 cm epoxy Robomussel	−30.1800	−71.4700
CLCOPT	Punta Talca	Coquimbo	Chile	8 cm epoxy Robomussel	−30.9200	−71.5000
GBENSP	Swanage Peveril Pt	England	United Kingdom	ibutton	50.6122	−2.1375
GBSCBK	Back	Scotland	United Kingdom	ibutton	56.4519	−5.4483
GBSCDS	Dunstaff	Scotland	United Kingdom	ibutton	56.4549	−5.4418
GBSCPP	Pump	Scotland	United Kingdom	ibutton	56.4531	−5.4428
IECLBH	Black Head	Clare	Ireland	ibutton	53.1542	−9.2648
IECLCQ	Coolsiva Quay	Clare	Ireland	ibutton	53.1428	−9.2261
IEGWBA	Ballynahown	Galway	Ireland	ibutton	53.2217	−9.5083
IEMYDO	Dooega	Mayo	Ireland	ibutton	53.9210	−10.0204
IEMYSA	Saula	Mayo	Ireland	ibutton	53.9535	−9.9271
MXBCAB	Punta Abreojo	Baja	Mexico	8 cm epoxy Robomussel, Tidbit	26.7262	−113.5450
MXBCBE	Baja Escorpion	Baja	Mexico	8 cm epoxy Robomussel	26.2377	−112.4786
MXBCBM	Bajamar	Baja	Mexico	8 cm epoxy Robomussel	31.9803	−116.7939
MXBCBT	Baja Tortugas	Baja	Mexico	8 cm epoxy Robomussel, Tidbit	27.6849	−114.9365
MXBCCR	Los Cerritos	Baja	Mexico	8 cm epoxy Robomussel, Tidbit	23.3288	−110.1811
MXBCER	Erendira	Baja	Mexico	8 cm epoxy Robomussel	31.3203	−116.4362
MXBCES	Esmeralda	Baja	Mexico	8 cm epoxy Robomussel	28.5168	−114.0724
MXBCOJ	Los Ojitos	Baja	Mexico	8 cm epoxy Robomussel	28.8823	−114.4238
MXBCPB	Punta Baja	Baja	Mexico	8 cm epoxy Robomussel	29.9497	−115.8134
MXBCPM	Punta Morro	Baja	Mexico	8 cm epoxy Robomussel	31.8614	−116.6678
NZAKAW	Anawhata	Auckland	New Zealand	8 cm epoxy Robomussel	−36.9170	174.4500
NZAKPK	Pakiri	Auckland	New Zealand	8 cm epoxy Robomussel	−36.2598	174.7531
NZCBBT	Box Thumb	Canterbury	New Zealand	8 cm epoxy Robomussel	−43.3506	172.7902
NZCMWW	Whau Whau Beach	Coromandel	New Zealand	8 cm epoxy Robomussel	−36.7789	175.7486
NZNLEB	Elliots Beach	Northland	New Zealand	8 cm epoxy Robomussel	−35.1148	173.9604
NZNLHH	Herekino Harbour	Northland	New Zealand	8 cm epoxy Robomussel	−35.2946	173.1571
NZNLMB	Maunganui Bluff	Northland	New Zealand	8 cm epoxy Robomussel	−36.7516	173.5695
NZWCWB	Woodpecker Bay	West Coast	New Zealand	8 cm epoxy Robomussel	−42.0190	171.2271
USCAAG	Alegria	California	USA	8 cm epoxy Robomussel, ibutton	34.4672	−120.2770
USCABD	Bodega Reserve	California	USA	8 cm epoxy Robomussel, ibutton	38.3185	−123.0740
USCABH	Boat House	California	USA	8 cm epoxy Robomussel	34.7188	−120.6088
USCACA	Cambria	California	USA	8 cm epoxy Robomussel	35.5400	−121.0929
USCACI	Bird Rock	California	USA	8 cm epoxy Robomussel	33.4514	−118.4861
USCACM	Cape Mendocino	California	USA	8 cm epoxy Robomussel	40.3480	−124.3650
USCACP	Coal Oil pt	California	USA	8 cm epoxy Robomussel, ibutton	34.4067	−119.8783
USCAFR	Fraser	California	USA	8 cm epoxy Robomussel	34.0627	−119.9192
USCAHS	Hopkins	California	USA	8 cm epoxy Robomussel, ibutton	36.6219	−121.9053
USCAJA	Jalama	California	USA	8 cm epoxy Robomussel, ibutton	34.4952	−120.4969
USCALL	Lompoc Landing	California	USA	8 cm epoxy Robomussel, ibutton	34.7191	−120.6089
USCALS	Lompoc South	California	USA	8 cm epoxy Robomussel, ibutton	34.7143	−120.6075
USCAPD	Piedras	California	USA	8 cm epoxy Robomussel	35.6658	−121.2867
USCAPR	Prisoners Harbor	California	USA	8 cm epoxy Robomussel	34.0204	−119.6866
USCATL	Trailer	California	USA	8 cm epoxy Robomussel	34.0517	−119.9032
USCATR	Terrace Point	California	USA	8 cm epoxy Robomussel	41.0621	−124.1493
USCAVD	Trinidad	California	USA	8 cm epoxy Robomussel	41.0621	−124.1493
USCAVL	Valley	California	USA	8 cm epoxy Robomussel	33.9837	−119.6658
USCAWL	Willows	California	USA	8 cm epoxy Robomussel	33.9618	−119.7549
USMADC	Dorothy Cove	Massachusetts	USA	4 cm shell robomussel	42.4238	−70.9208
USMAFR	Forest River	Massachusetts	USA	4 cm shell robomussel	42.4976	−70.8868
USMANP	East Point	Massachusetts	USA	4 cm shell robomussel	42.4200	−70.9022
USMAOP	Obear Park	Massachusetts	USA	4 cm shell robomussel	42.5451	−70.9014
USMAPH	Pumphouse	Massachusetts	USA	4 cm shell robomussel	42.4168	−70.9067
USORBB	Boiler Bay	Oregon	USA	8 cm epoxy Robomussel, ibutton	44.8306	−124.0601
USORCA	Cape Arago	Oregon	USA	8 cm epoxy Robomussel	43.3066	−124.4024
USORFC	Fogarty Creek	Oregon	USA	8 cm epoxy Robomussel	44.8373	−124.0585
USORSH	Strawberry Hill	Oregon	USA	8 cm epoxy Robomussel, ibutton	44.2499	−124.1136
USSCOL	Oyster Landing	South Carolina	USA	8 cm Shell Robomussel	33.3495	−79.1888
USWACC	Colins Cove	Washington	USA	8 cm epoxy Robomussel, ibutton	48.5494	−123.0060
USWACP	Cattle Point	Washington	USA	8 cm epoxy Robomussel, ibutton	48.4514	−122.9618
USWALB	Landing Beach	Washington	USA	8 cm epoxy Robomussel	48.3938	−124.7355
USWASD	Strawberry Point	Washington	USA	8 cm epoxy Robomussel	48.3914	−124.7384
ZAECCA	Cape St Francis	Eastern Cape	South Africa	8 cm epoxy Robomussel	−34.2095	24.8374
ZAECJO	Jongensfontein	Eastern Cape	South Africa	8 cm epoxy Robomussel	−34.4198	21.3574
ZAECKB	Kidd's Beach	Eastern Cape	South Africa	8 cm epoxy Robomussel	−33.1475	27.7033
ZAECKE	Kenton-on-sea	Eastern Cape	South Africa	8 cm epoxy Robomussel	−33.6942	26.6678
ZAECMB	Morgans Bay	Eastern Cape	South Africa	8 cm epoxy Robomussel	−32.7111	28.3396
ZAECSK	Skoenmakerskop	Eastern Cape	South Africa	8 cm epoxy Robomussel	−34.0408	25.5333
ZAKNBA	Ballito	KwaZulu-Natal	South Africa	8 cm epoxy Robomussel	−29.4838	31.2592
ZAKNPE	Port Edward	KwaZulu-Natal	South Africa	8 cm epoxy Robomussel	−31.0480	30.2302
ZANCHO	Hondeklipbaai	Northern Cape	South Africa	8 cm epoxy Robomussel	−30.3061	17.2707
ZAWCBT	Brenton	Western Cape	South Africa	8 cm epoxy Robomussel	−34.0752	23.0239
ZAWCDO	Doringbaai	Western Cape	South Africa	8 cm epoxy Robomussel	−31.8004	18.2298
ZAWCKB	Keurboom	Western Cape	South Africa	8 cm epoxy Robomussel	−34.0050	23.4553
ZAWCPB	Plettenberg	Western Cape	South Africa	8 cm epoxy Robomussel	−34.0616	23.3798
ZAWCPN	Paternoster	Western Cape	South Africa	8 cm epoxy Robomussel	−32.8019	17.8983
ZAWCRO	Robberg	Western Cape	South Africa	8 cm epoxy Robomussel	−34.1022	23.3808
ZAWCYZ	Yzerfontein	Western Cape	South Africa	8 cm epoxy Robomussel	−33.3467	18.1531

**Table 2:** Data descriptors.

**Parameter**	**Description**	**Unit**
Logger type	Type of biomimic or logger	Text
Site Code	6-character site identification code (1st 2-characters: country; 2nd 2-characters: state; 3rd 2-characters: site)	Text
Site Name	Name of the site	Text
Region	Name of the region or jurisdiction	Text
Country	Name of the country	Text
Latitude	Latitude of the site	Decimal degree
Longitude	Longitude of the site	Decimal degree
Zone	Intertidal zone (low, lower mid, mid, upper-mid, upper)	Text
Substrate	Additional characteristics: muddy intertidal, rocky intertidal	Text
Wave Exposure	Wave exposure of logger (exposed or protected)	Text
